# Refractory dissecting cellulitis of the scalp treated with risankizumab: 2 case reports

**DOI:** 10.1016/j.jdcr.2023.10.011

**Published:** 2023-10-29

**Authors:** Khalid Nabil Nagshabandi, Abdulaziz Alsalhi, Duaa Alahmadi, Sara Almesfer, Abdulmajeed M. Alajlan

**Affiliations:** a Department of Dermatology, College of Medicine King Saud University and King Saud University Medical City, Riyadh, Saudi Arabia; b College of Medicine, Al-Rayan University, Medina, Saudi Arabia; c College of Medicine, Dar Al-Uloom University, Riyadh, Saudi Arabia

**Keywords:** biologics, DCS, dissecting cellulitis, IL-23 inhibitors, risankizumab

## Introduction

Dissecting cellulitis of the scalp (DCS), often called Hoffmann disease, is a rare chronic inflammatory skin condition that affects mainly the vertex and occipital scalp. It clinically presents with perifollicular sterile pustules, boggy fluctuant nodules, keloids, abscesses, and sinus tracts before evolving into scarring alopecia.^1^ Dissecting cellulitis is a rare and aggressive form of scalp folliculitis that commonly affects males and African Americans. DCS is a debilitating condition that can significantly impact the quality of life of those affected and can result in severe psychological distress.^2^ Studies suggest that topical and systemic antibiotics, isotretinoin, and intralesional corticosteroids are the mainstay first-line treatment options, exhibiting varying degrees of efficacy in managing active lesions, as they are only effective in managing mild limited disease and patients are more prone to frequent relapses.^3^ There is no known cure for DCS, and patients may require lifelong management to prevent flares.^2^ Immunosuppressive agents such as tumor necrosis factor inhibitors have been used to manage DCS.^2^ An interleukin (IL) 17A inhibitor (secukinumab), has been reported in successfully treating DCS.^4^ We present 2 cases of risankizumab (IL-23 inhibitor), used to successfully treat recalcitrant DCS. Our report is the second of its kind that has exclusively used risankizumab in managing this disease. To the best of our knowledge, there are only 3 case reports of using IL-23 inhibitors (guselkumab, risankizumab, and tildrakizumab) in refractory DCS, one of which was given to a patient with concurrent hidradenitis suppurativa.^5^, ^6^, ^7^ And none of the studies reported using the Dermatology Life Quality Index in assessing the patient’s quality of life before and after biologic therapy, as we are the first to utilize this tool before and after using an IL-23 inhibitor.

## Case synopsis

### Case 1

**Fig 1:**
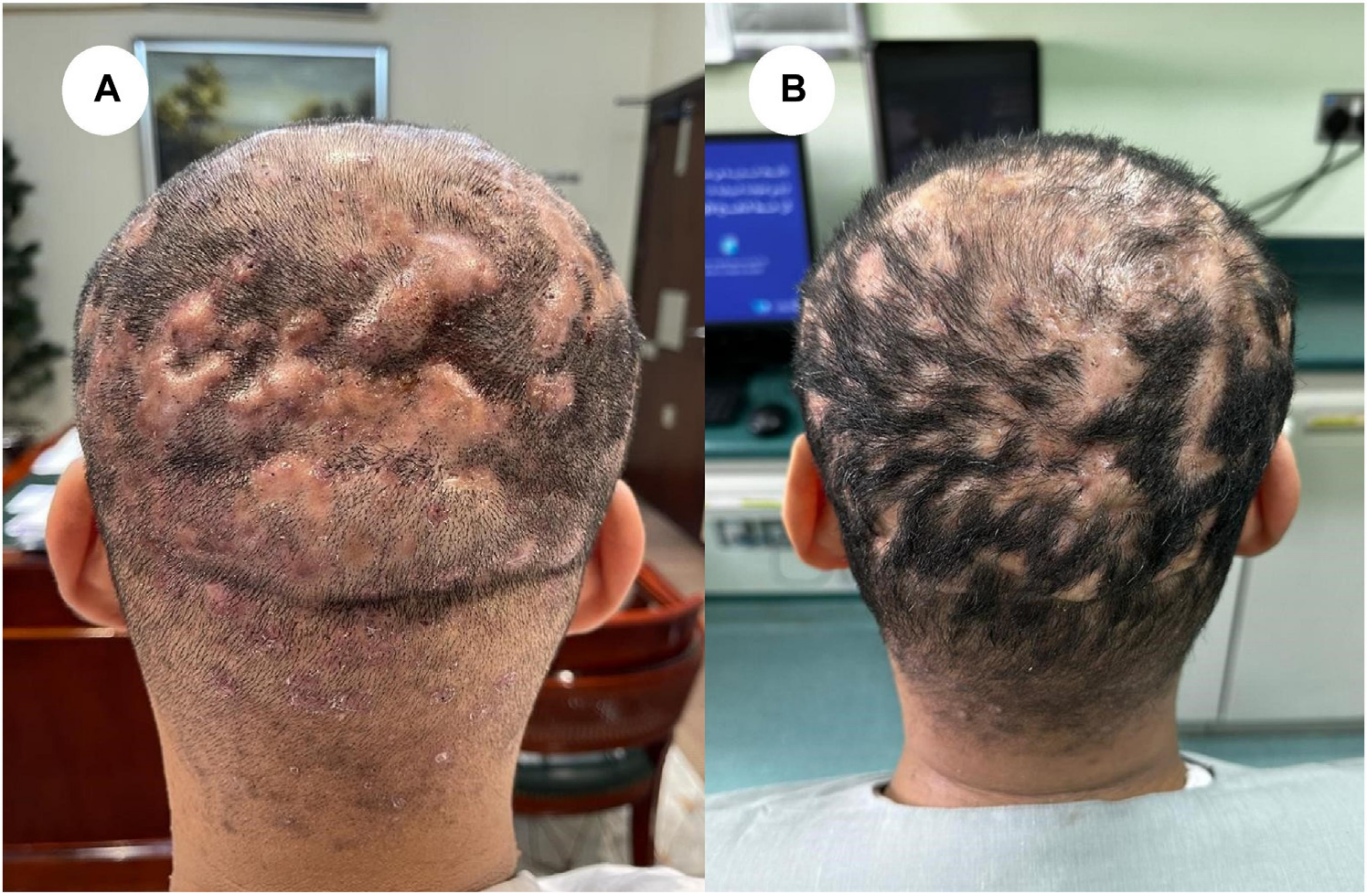
**A,** Erythematous indurated nodules over multiple regions of the scalp. **B,** After receiving 5 doses of risankizumab therapy, no new lesions or draining nodules appeared, and there were signs of hair regrowth.

**Fig 2:**
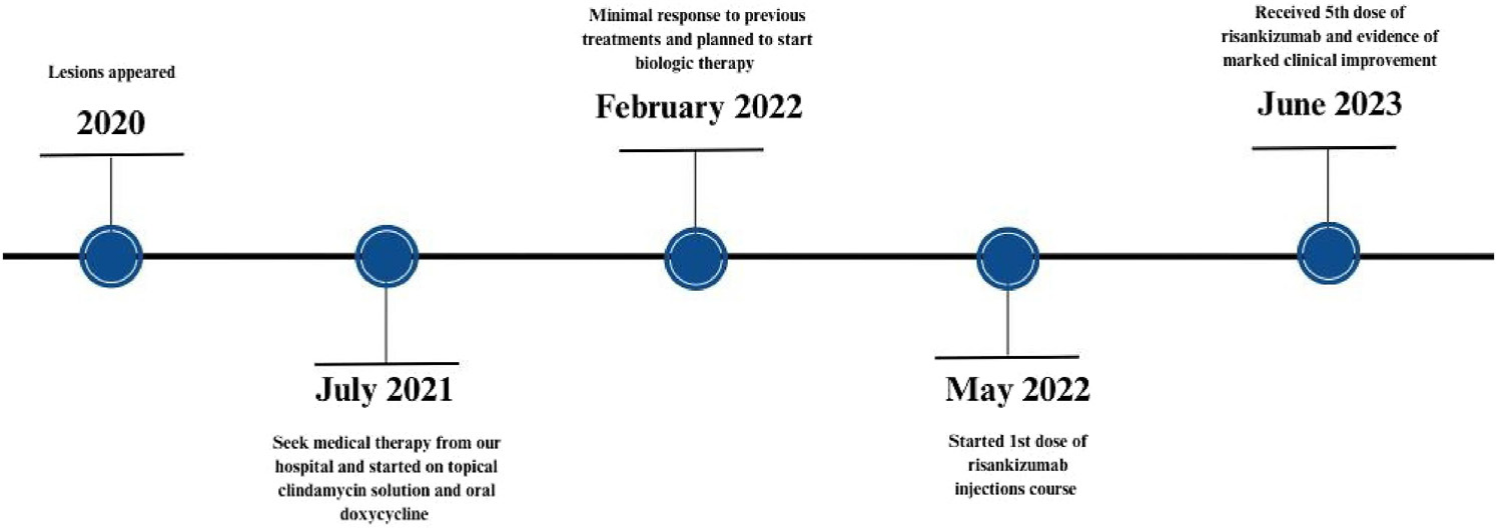
Case 1 timeline of events.

A 26-year-old male smoker, known to have type 2 diabetes mellitus on both insulin and metformin 500 mg and with a past surgical history significant for sleeve gastrectomy, presented to the dermatology outpatient clinic complaining of painful itchy lesions and hairless patches over the scalp for 2 years. Clinical examination showed multiple erythematous draining nodules with a few pustules over the scalp and scarring alopecia patches—clinical characteristics suggestive of dissecting cellulitis. He was prescribed topical clindamycin 1% solution and oral doxycycline 100 mg with minimal response after 6 months. The patient was then started on risankizumab in-clinic injection every 3 months. He reported an improvement of roughly 70% by the fifth dose of risankizumab and after 13 months of therapy. The lesions revealed signs of clinical remission (Fig 1). Timeline for the case (Fig 2).

### Case 2

**Fig 3:**
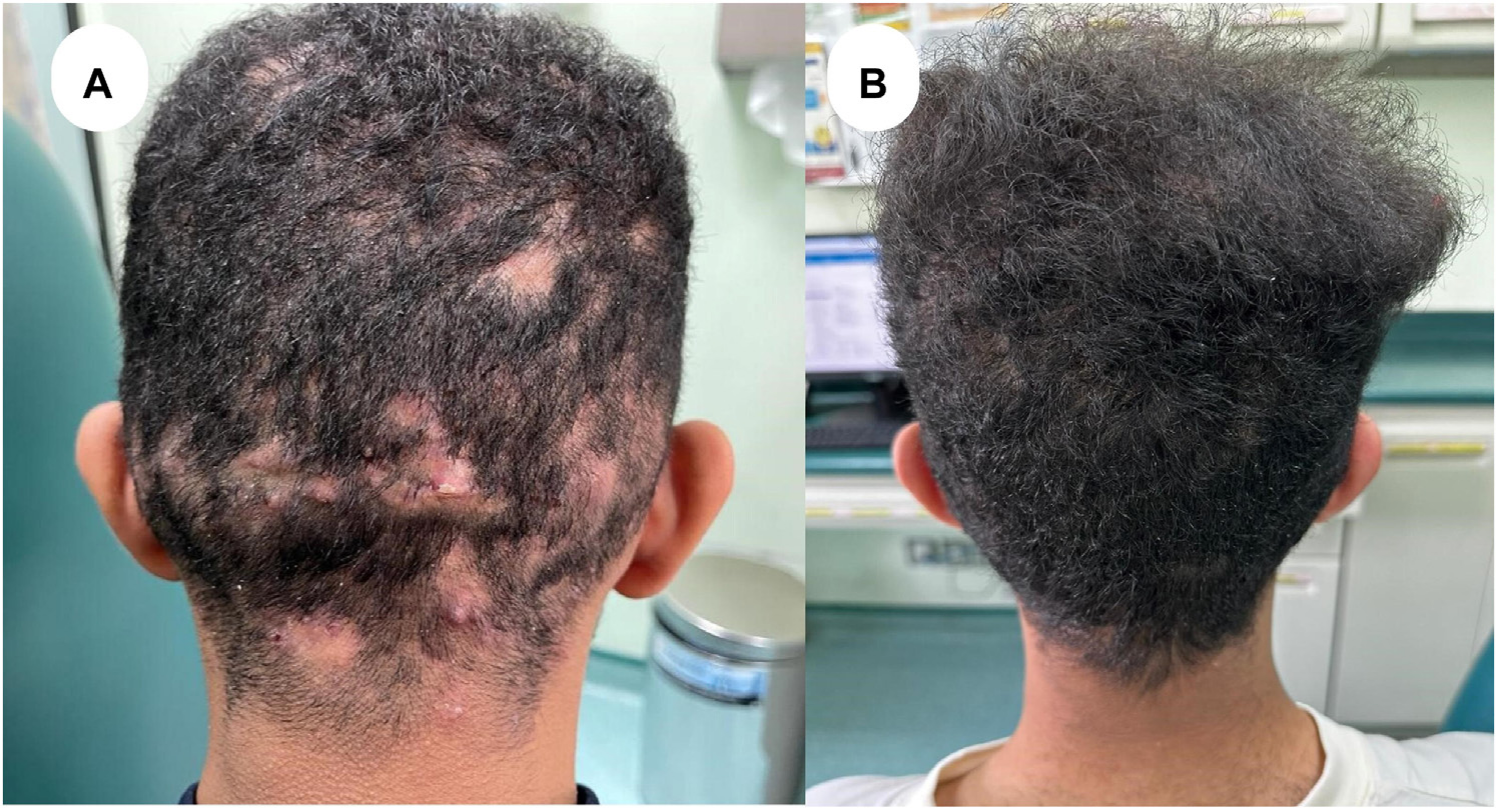
**A,** Erythematous indurated nodules mainly over the occipital scalp. **B,** After receiving 3 doses of risankizumab therapy, there was no evidence of new lesions, purulent drainage, or signs of hair regrowth.

**Fig 4:**
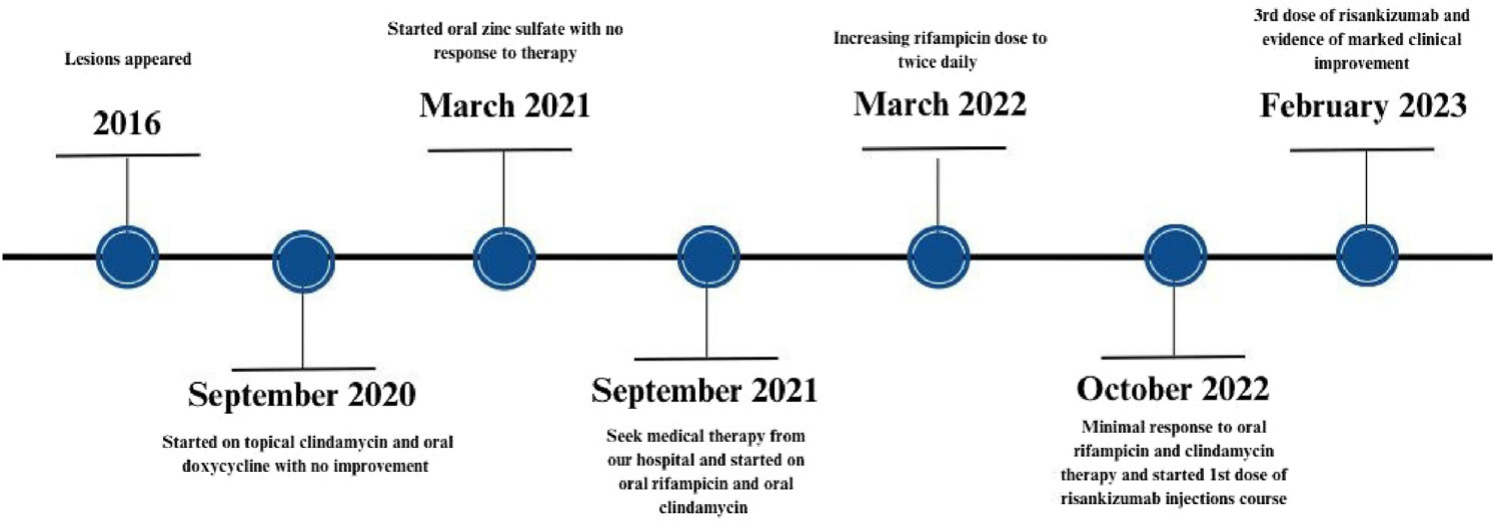
Case 2 timeline of events.

A medically free 17-year-old boy presented to our clinic with a biopsy that confirmed dissecting cellulitis of the occipital scalp for 5 years. The lesions started as painful erythematous nodules, followed by hair loss. Clinical examinations revealed a few erythematous nodules with hair regrowth mainly over the occipital scalp and crown and a solitary alopecic patch over the right temporal area. A dermatoscopy of the scalp showed miniaturized hair with few yellowish globules. He previously sought medical therapy from outside hospitals where he received 6 months of topical clindamycin solution and oral doxycycline 100 mg with no improvement, then 6 months of oral zinc sulfate. However, the disease progressed despite therapy. The management plan was to discontinue any current medication and start him on oral clindamycin 150 mg, oral rifampicin 300 mg, and continue applying topical minoxidil 5%. Rifampicin dose was increased to 600 mg daily after a 6-month follow-up. After almost 1 year of receiving rifampicin, the patient presented with worsening skin lesions. Physical examination showed multiple boggy scarring alopecic patches with surrounding pustules over the occipital scalp. The patient was then started on risankizumab injection course, and topical clindamycin 1% solution applied daily. He reported improvement by the third dose of risankizumab and after 4 months of therapy, the lesions showed signs of clinical remission (Fig 3). Timeline for the case (Fig 4).

## Discussion

Limited data is available regarding the treatment of DCS. There are no clear guidelines to properly manage the disease, and no standard therapy exists despite having a previous study recommending a treatment approach for refractory DCS.^2^ Determining the best management plan for DCS is frustrating. Multiple treatment options are available with varying degrees of efficacy, including topical clindamycin, oral zinc, isotretinoin, dapsone, doxycycline, minocycline, sulfa antibiotics, rifampicin, metronidazole, and glucocorticosteroids. Biologic therapies include anti–tumor necrosis factor and IL-23 inhibitor risankizumab. Laser hair removal and surgical excision can be considered in unresponsive cases.^2^ Despite harboring numerous therapeutic modalities, relapses and frequent flares are common and may cause further psychological distress for the patient. Both of our patients (case 1 and case 2) failed their previous therapy and exhibited worsening skin lesions after multiple follow-ups. The Dermatology Life Quality Index is a 10-question questionnaire used to measure the impact of skin diseases on the quality of life of the patient.^8^ We have utilized this tool to assess the impact of DCS on the patient’s quality of life before and after risankizumab therapy. Case 1 and case 2 showed a score of 21 out of 30 (extremely large effect on patient’s quality of life) and 14 out of 30 (very large effect on patient’s life), respectively, before being injected with risankizumab, in comparison to a score of 10 out of 30 (small effect on patient’s life) and 1 out of 30 (no effect on patient’s life), respectively, after risankizumab injections. The transitioning of therapy to risankizumab, an IL-23 inhibitor, resulted in marked clinical and psychosocial improvement.

Risankizumab use has expanded to successfully manage other dermatological conditions, such as hidradenitis suppurativa often off-label.^9^ DCS and hidradenitis suppurativa are clinically interrelated and can often coexist as a follicular occlusion triad.^10^ This could indicate the reason why our patients responded favorably to risankizumab therapy. Risankizumab, therefore, may be an excellent choice for treating recalcitrant DCS.

## Conflicts of interest

None disclosed.
